# FSPLO: a fast sensor placement location optimization method for cloud-aided inspection of smart buildings

**DOI:** 10.1186/s13677-023-00410-0

**Published:** 2023-03-06

**Authors:** Min Yang, Chengmin Ge, Xiaoran Zhao, Huaizhen Kou

**Affiliations:** 1 Chongqing Vocational Institute of Engineering, Chongqing, China; 2 Shandong Provincial University Laboratory for Protected Horticulture, Weifang University of Science and Technology, Weifang, China; 3 Chinese Academy of Education Big Data, Beijing, China; 4 School of Computer Science and Engineering, Nanjing University of Science and Technology, Nanjing, China; 5 School of Computer Science, Qufu Normal University, Rizhao, China

**Keywords:** Cloud computing, Smart building, Sensor location optimization, Locality-sensitive hashing, High efficiency

## Abstract

With the awakening of health awareness, people are raising a series of health-related requirements for the buildings they live in, with a view to improving their living conditions. In this context, BIM (Building Information Modeling) makes full use of cutting-edge theories and technologies in many domains such as health, environment, and information technology to provide a new way for engineers to design and build various healthy and green buildings. Specifically, sensors are playing an important role in achieving smart building goals by monitoring the surroundings of buildings, objects and people with the help of cloud computing technology. In addition, it is necessary to quickly determine the optimal sensor placement to save energy and minimize the number of sensors for a building, which is a de-trial task for the cloud platform due to the limited number of sensors available and massive candidate locations for each sensor. In this paper, we propose a Fast Sensor Placement Location Optimization approach (FSPLO) to solve the BIM problem in cloud-aided smart buildings. In particular, we quickly filter out the repeated candidate locations of sensors in FSPLO using Locality Sensitive Hashing (LSH) techniques to maintain only a small number of optimized locations for deploying sensors around buildings. In this way, we can significantly reduce the number of sensors used for health and green buildings. Finally, a set of simulation experiments demonstrates the excellent performance of our proposed FSPLO method.

## Introduction

The continuous economic development of society has significantly improved people’s living conditions and changed their perception of life. In this situation, high-quality life has surpassed the survival needs as an important goal for human-being [1–3] to pursue. In addition, with the widely spread of COVID-19 pandemic and other epidemic diseases, there is an increasing emphasis on healthy living [4, 5]. In other words, people are now willing to pay more attentions to healthcare than ever before, which promotes the quick development of health business or industry and as a result, brings more development opportunities for a series of health-related domains such as smart medicine, smart home, smart sports and smart building [6, 7].

As an important place where people live and stay, buildings are receiving increasing attention from both academia and industry due to their natural advantages for improving healthy living. To achieve this goal, the concept of smart buildings is proposed by introducing sensors and cloud platforms to monitor and analyze real-time environmental conditions (e.g. weather, temperature, humidity, sunlight, wind, etc.) of buildings to understand the specific building environment as quickly and accurately as possible [8, 9]. After obtaining detailed monitoring data, corresponding measures can be taken to cope with the unexpected changes of the built environment. For example, when the cloud platform reports a heavy rainfall in the next few hours after analyzing the data obtained through humidity sensors, windows are closed automatically to prevent rain from entering the building. Therefore, sensors have become an integral part of smart buildings as occupants’ requirements for healthy living continue to increase [10]. However, the diversity of detection data and the range of sensor roles dictate that deploying a large number of sensors with diverse functions is necessary if a building’s real-time environmental conditions are to be comprehensively detected. However, this approach requires deploying a large number of sensors, which is not economical enough, and increases the operational burden of the central server [11–13]. In this case, it becomes necessary to optimize the deployment location of sensors in the building to reduce the number of sensors. In general, two conditions must be met for an optimal sensor deployment strategy: (1) the sensors should monitor and collect as much information as possible to analyze more valuable information through the cloud platform; (2) the sensors should have the characteristic of “the fewer the better” to reduce the cost of sensors and the burden on the cloud platform servers. However, they are often in conflict with each other, as fewer sensors can only monitor and collect less information about the building. Therefore, developing an optimal solution that meets both of these conditions is a big challenge.

In view of this challenge, a hash-based fast sensor placement location optimization method for BIM in cloud-aided smart building is proposed in this paper. Specifically, through hashing the environmental monitoring data obtained from sensors deployed at different locations, the cloud platform can discover those sensors with similar monitoring data. In this way, through eliminating redundant sensors that have similar data deployed in different locations, we can reduce the number of sensors that need to be maintained.

In general, the major contributions and innovations of our paper are three-fold.

1. We recognize the importance and research significance of sensor saving in creating a green and healthy building through various sensor deployment location optimization strategies in cloud-aided BIM. For a building, the deployed sensors could be reduced considerably.
2. We model the sensor deployment location optimization problem in BIM-driven smart building as a similar sensor discover problem through observing the historical monitoring data by sensors deployed in different locations around the building. Through recognizing the similar sensors by a hash process in the cloud platform, partial unnecessary sensors could be saved, which is beneficial to constructing healthy, green and economical buildings for human-beings.
3. To validate the feasibility of the proposed sensor deployment location optimization method, we have designed a set of experiments to simulate the concrete effectiveness and efficiency of our proposal. Reported results prove the advantages of the proposal.

The reminder of this paper is structured as below. Related literatures associated with cloud-aided smart building are investigated and summarized in Section 2. A motivating example is presented in Section 3 to highlight the research background and significance of our study. The proposed sensor deployment location optimization method is introduced in Section 4. In Section 5, massive experiments are enacted and tested to prove the feasibility of our proposal. At last, in Section 6, we summarize the whole paper and point out the future research directions in the upcoming study.

## Related work

### Energy management in cloud-aided building

In [14], the authors propose a portable sensor network platform-BiB, which is very easy to be deployed in any building environment. BiB is low-cost and battery-powered sensors that are small and light enough to be unobtrusively installed in office spaces and connected to a cloud platform. BiB sensors can collect rich environmental parameters and can be extended to measure other parameters, such as carbon dioxide. BiB has monitored thousands of buildings to identify the most effective ways to improve the building environment in terms of energy efficiency via big data analysis in cloud platform. In [15], aiming at the problem of highly unreliable sensors and cloud platform in the practical application of energy intelligent buildings, the author proposes a hierarchical probabilistic framework for occupation-based control of intelligent buildings. The method uses hierarchical linkage, where each layer deals with different aspects of the occupancy detection problem in a probabilistic manner. And the performance of the entire system can be improved by propagating uncertainty through each layer rather than the standard hard decision results. Compared to baseline measurements, the method achieved energy savings of up to 30% while maintaining user comfort. In [16], the authors propose a new energy saving strategy based on BEMS. In this paper, firstly, the concept of BEMS monitoring is proposed, and the communication protocol to cloud platform of BEMS is discussed. Then, the implicit occupancy detection method is used to calculate the number of people in the building. This approach is largely based on existing non-energy IT infrastructure and has a low cost and high accuracy in data collection compared to traditional methods. Finally, an energy saving strategy based on occupancy data is proposed. The simulation results show that the proposed method is effective in reducing building energy consumption. These methods can effectively collect the data of intelligent buildings and make use of it. However, these methods do not take into account the privacy implications of the data collected in smart buildings, and the risk of leakage increases if the data is used without protection.

### Data processing in smart building

In [17], faced with more and more data and the relationship between complex data points, the authors propose a simple, economical and privacy-protecting method to extract occupancy information. A virtual residential sensor is developed by aggregating semantic knowledge, motion sensor data and residential entrance data. This approach can be replicated in all building environments described in a similar way, where movement information is collected, monitoring spaces have clear boundaries, and occupancy information can be useful in different application cases. While predictive occupancy models or expensive sensing alternatives have been used for similar purposes, the solution is simple, inexpensive, replicable, and easy to implement in existing buildings. In [18], the authors propose a candidate wireless sensor network for indirect occupancy measurement of cloud-aided intelligent buildings. It can interact with its local environment to provide the necessary information and work with contextual information. To provide the necessary environmental monitoring, the prototype has three sensor units for measuring carbon dioxide, temperature and humidity. The prototype is placed in a complex indoor environment to collect data for a week. The data is then analyzed by cloud platform to examine potential correlations between sensor data and occupancy information. Experimental results on real world dataset show that CO2 can be used for indirect occupancy measurement. In [19], the authors focus on intelligent buildings and “Internet of Things”. Based on similar techniques and systems proposed by other researchers in this field, the authors propose an efficient intelligent building network architecture composed of sensor devices. These devices are smart enough to transmit the massive data generated by “Internet of Things” [20] to the Internet via routers, thus providing effective solutions for smart cities. In [21], in order to deal with outliers in the data collected by sensors, this paper proposes an outlier detection and recovery method using artificial neural network (ANN), which is used to judge whether the temperature measured by sensors in sensor networks is an outlier. In addition, an optimized neural network model is proposed to improve the detection accuracy when the ambient temperature varies greatly. Experiments show that the ANN model can effectively predict the temperature of wireless sensor network system in smart buildings. In [22], the clustering idea is considered a classical and effective mechanism for providing reliable and energy efficient data transmission in smart building systems in order to process and transmit multimedia data efficiently. In the clustering, the whole perception region is divided into several small regions, and a designated node CH is assigned to each small region for energy optimization. In this paper, the authors combine Grenade Explosion Method (GEM) and Cauchy operator in CH selection process to improve the exploration and utilization of the Artificial Bee Colony (ABC) algorithm. A new IABCOCT algorithm is derived. This algorithm avoids the possibility of limiting the search process to a specific area and has high computational efficiency in the search process. While these methods are effective in making buildings “smarter”, they do not optimize sensor placement, which leads to excessive data collection and an increased burden on central servers.

### Security and privacy in cloud data processing

In [23], the authors put forward a flow counting algorithm based on low resolution thermal images to avoid privacy problems in view of the possible challenges such as user privacy, communication limitation and sensor computing capability in cloud environment. At the same time, the scheme is also suitable for wireless sensor networks based on “Internet of Things” platform. This method can effectively solve the privacy problem in wireless sensor networks, and develop a practical occupation monitoring system for cloud-aided intelligent buildings. In [24], aiming at the problem of data sensitivity in smart cloud environment, the authors propose a new intrusion detection system IDS that can provide a perfect security scheme for wireless sensor networks in intelligent cloud environment. The device is also a sensor, with more power and memory space, adopts the computing capabilities of sensors in a network, and achieves network security by filtering all incoming and outgoing communications. With the above analyses and summarization, we can conclude that existing research literatures about smart building are still faced a wide range of difficulties and challenges especially when the sensors deployment and sensor-cloud transmission costs are expensive [25–27] and energy-consuming [28–30]. Considering these challenges, we put forward a novel sensor deployment location optimization method for smart buildings to minimize the number of sensors employed for building monitoring.

## Motivation

**Fig. 1:**
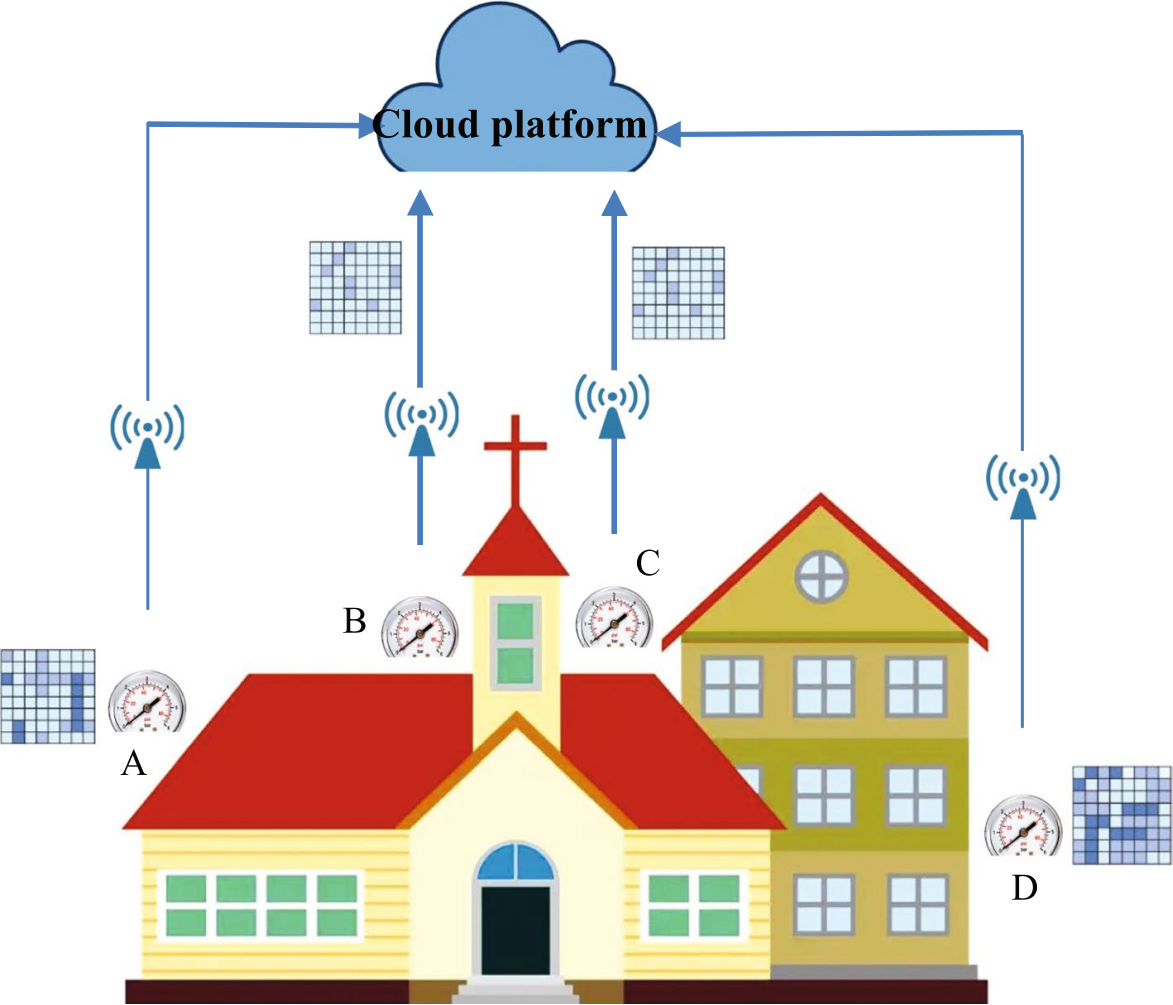
Cloud sensor-based smart building: an example scenario and challenge

A real-world example is introduced in this section to better clarify the research motivation of this paper. As Fig. 1 depicts, there are four sensors deployed around a house, i.e., A, B, C and D, each of which can help the house owner to monitor and collect the real-time environment information of the house (e.g., temperature, humidity, light, etc) and transmitted to a central cloud platform. Here, each sensor can collect a set of environmental signals of the house, which are organized in a time-aware matrix form (each row denotes an environmental criterion or indicator and each column represents a time point). However, the range of data collected by these sensors overlaps, which leads to sensor waste and additional deployment costs. This high cost brings a great challenge for the house owner to build his house in a green, healthy and economic way. In view of this challenge, a fast sensor placement location optimization method (named FSPLO) is proposed in this paper for BIM in health-driven smart building. In concrete, in FSPLO, we utilize the Locality-Sensitive Hashing (LSH) technique [31] to quickly filter out the repeated candidate locations for sensors to maintain only a small number of locations for deploying sensors connecting the cloud platform. With optimization, the number of sensors in a room can be reduced to three, which not only reduces deployment costs, but also does not affect the collection of environmental information.

## Method

**Fig. 2:**
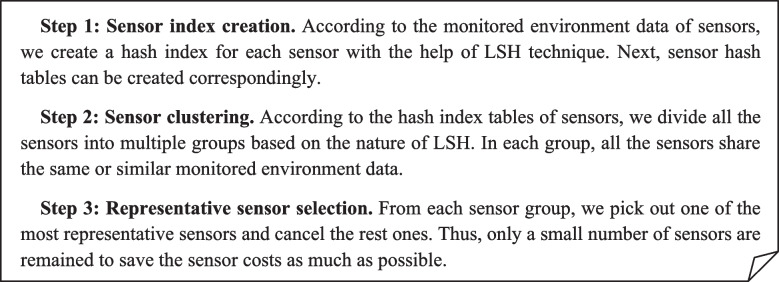
Main procedure of the proposed FSPLO method

In this section, we provide a detailed overview of our proposed approach, called FSPLO. In general, our proposed FSPLO method consists of the following three steps presented in Fig. 2. Firstly, for each sensor deployed around a building and connected to a cloud platform, we construct its hash index based on LSH technique. Second, we cluster the multiple sensors corresponding to the building into different groups based on their respective index values. Third, for each sensor group, we pick one representative sensor and deploy it on the building. The three steps would be specified in detail in the following content of this section.

### Sensor index creation

Here, for more formalization, we assume that there are *M* candidate sensors deployed around a building (e.g., *M* = 4 in the example scenario of Fig. 1), denoted by $Set_M$ = {$S_1$, $S_2$, $\cdots$, $S_M$}; each sensor can monitor *N* environment indicators or criteria (e.g., temperature, humidity, light, and so on), represented by $Set_N$ = {$I_1$, $I_2$, $\cdots$, $I_N$}; at a certain time point, the $I_j$ ($1 \le j \le N$) value monitored by the sensor $S_i$ ($1 \le i \le M$) is denoted by $v_i,j$. Thus, at a certain point, the monitored environment data by all the sensors can be formalized with a matrix *V* in (1). For example, the first row of matrix *V*, i.e., $\phi _1 = (v_{1,1}, \cdots , v_{1,N})$ represents the first sensor, i.e., $S_1$’s monitored data over *N* environment indicators $I_1$, $I_2$, $\cdots$, $I_N$.

1 $$\begin{aligned} V=\begin{array}{c} {{S}_{1}} \\ \vdots \\ {{S}_{M}} \\ \end{array}\overset{\begin{array}{ccc} I_1 &{} \cdots &{} I_N \\ \end{array}}{\left[ \begin{array}{ccc} v_{1,1} &{} \ldots &{} v_{1, N} \\ \vdots &{} \cdots &{} \vdots \\ v_{M, 1} &{} \cdots &{} v_{M, N} \\ \end{array} \right] }\ \end{aligned}$$

To reduce the number of sensors deployed on a building, we need to find out the sensors which have been monitoring the same or similar data over all the *N* environment indicators {$I_1$, $I_2$, …, $I_N$}. However, such comparisons among different sensors (typically, in the form of vectors such as vector ($v_{1,1}$, …, $v_{1,N}$) corresponding to sensor $S_1$ in (1)) are often time-consuming due to the complex matrix computation. Furthermore, with time elapsing, the monitored environment data of sensors are also variable, which further increase the time complexity for comparing two sensors. To minimize the time complexity, we introduce LSH technique to realize fast and time-efficient sensor comparison and reduction.

According to the analysis in the above paragraph, each row *i* of matrix *V* is corresponding to a vector $\phi _1$ = ($v_{1,1}$, …, $v_{1,N}$). Next, we randomly produce a vector *A* = ($a_1$, …, $a_N$) where each entry $a_j$ ($1 \le j \le N$) satisfies the condition in (2). Afterwards, we execute a dot product operation between vectors $\phi _1$ and *A*, after which we obtain a concrete value $b_i$ as formalized in (3). Next, we execute the conversion or transformation presented in (4) to build a mapping from $b_i$ to $c_i$ ($1 \le \textit{i} \le \textit{M}$). More concretely, if $b_i$ is positive, then $c_i$ would be equal to 1; otherwise, $c_i$ would be equal to 0. The more intuitive explanation of the conversion from $b_i$ to $c_i$ is: we randomly pick out a hyperplane *A* to divide the whole space into two parts; if point $b_i$ is above the hyperplane *A*, then we record $c_i$ = 1; otherwise, if point bi is below the hyperplane *A*, then we record $c_i$ = 0. Then according to the above analysis, if two points $b_{i1}$ and $b_{i2}$ are both located on the same side of the hyperplane *A*, then $b_{i1}$ and $b_{i2}$ are more likely similar.

2 $$\begin{aligned} a_j = rand(-1,1)\quad (1 \le {j} \le {N}) \end{aligned}$$

3 $$\begin{aligned} b_i=\phi _i \circledcirc A=\sum \limits _{j=1}^{N}v_{i,j}*a_{j} \end{aligned}$$

4 $$\begin{aligned} c_i = \left\{ \begin{array}{c} 1 \quad if \ b_i > 0\\ 0 \quad if \ b_i \le 0 \end{array}\right. \end{aligned}$$

However, the above simple mapping from $b_i$ to $c_i$ is often based on probability. In other words, it is not accurate to evaluate whether two points are similar or not if we execute the mapping operations in (2)-(4) only once. Therefore, to achieve higher similarity evaluation accuracy, we repeat the operations in (2)-(4) multiple times (more formally, we assume *X* times (*X* is a positive integer, i.e., *X* > 1)). Thus, we can get a series of *A*, i.e., { $A_1$, …, $A_X$ }; correspondingly, according to (3)-(4), we can get a series of *b* and *c* values, i.e., { $b_1$, …, $b_X$ } and { $c_1$, …, $c_X$ }, respectively. After that, each vector $\phi _1$ corresponding to sensor $S_i$ is assigned a 0-1 vector $C_i$ = ( $c_1$, …, $c_X$ ). According to the LSH rule, $C_i$ can be used to represent vector $\phi _1$; in other words, $C_i$ is the index of $\phi _1$. Here, please note that the above index generation step can be done in an offline way; therefore, the time complexity of this step is approximately O(1).

### Sensor clustering

According to Step 1, we can assign an index for each of the *M* sensors in (1). More formally, the matrix *V* in (1) is updated to be a vector $V^*$ presented in (5). Then according to LSH, sensor $S_i$ and $S_j$ are similar if and only if their respective hash values are equal, i.e., $C_i$ = $C_j$ (we formalize this conclusion in (6)). However, as we analyzed in Step 1, LSH is a probability-based similar object search technique. Therefore, the condition in (6) is too strict for evaluating whether two sensors are similar very accurately.

5 $$\begin{aligned} {{V}^{*}}=\begin{array}{c} {{S}_{1}} \\ \vdots \\ {{S}_{M}} \\ \end{array}\left[ \begin{array}{c} {{C}_{1}} \\ \vdots \\ {{C}_{M}} \\ \end{array} \right] =\begin{array}{c} {{S}_{1}} \\ \vdots \\ {{S}_{M}} \\ \end{array}\left[ \begin{array}{ccc} c_{1,1} &{} \cdots &{}c_{1,N} \\ \vdots &{} \cdots &{} \vdots \\ c_{M,1} &{} \cdots &{} c_{M,N} \\ \end{array} \right] \end{aligned}$$

6 $$\begin{aligned} S_i \ and \ S_j \ are \ similar \ if \ C_i = C_j \end{aligned}$$

Considering this drawback, we repeat Step 1 multiple times to reduce the uncertainty in similar sensor evaluation. In concrete, we repeat (2)-(5) Y times offline and through which we can obtain Y index values for each sensor $S_1$, $S_2$, …, $S_M$. More formally, vector in (5) is extended to be a new matrix as presented in (7). Here, $V^\#$ is a $M*Y$ matrix and each row denotes the *Y* index values of a sensor. Then through the *Y* columns of $V^\#$, we can relax the search condition previously formalized in (6). Concretely, we can evaluate whether two sensor $S_i$ and $S_j$ are similar through the new judgment condition in (8). Please note that the approximate neighbor search condition in (8) is a kind of relaxation of the condition in (6). More intuitively, if the index values of two sensors are equal in any column of matrix $V^\#$, then we can deem that these two sensors are probably similar with each other. In this way, we can reduce the uncertainty of similar neighbor evaluation brought by the nature of LSH technique. Next, all the similar sensors are divided and put into different groups $G_1$, … , $G_K$ with diverse environment monitoring functions.

7 $$\begin{aligned} {{V}^{*}}=\begin{array}{c} {{S}_{1}} \\ \vdots \\ {{S}_{M}} \\ \end{array}\overset{\begin{array}{ccc} 1 &{} \cdots &{} Y \\ \end{array}}{\left[ \begin{array}{ccc} {{C}_{1,1}} &{} \ldots &{} {{C}_{1,Y}} \\ \vdots &{} \cdots &{} \vdots \\ {{C}_{M,1}} &{} \cdots &{} {{C}_{M,Y}} \\ \end{array} \right] }\ \end{aligned}$$

8 $$\begin{aligned} S_i \ and \ S_j \ are\ similar\ if \ C_{i,y} = C_{j,y} \quad (1 \le y \le Y) \end{aligned}$$

### Representative sensor selection

In Step 2, we have divided all the M sensors into different groups $G_1$, $G_2$, …, $G_K$ based on their index values and the evaluation condition in (8). Each group consists of multiple sensors whose monitored environment data of a building are similar. In this situation, the effect that all the sensors in an identical group take is close. Therefore, it is not necessary to maintain so many sensors with repeated effect or functions. In other words, we can eliminate most of the repeated sensors from each of the sensor groups derived in Step 2.

Next, we introduce how to find out the best sensor $S_{best}$ among multiple candidates {$S_1$, $S_2$, …, $S_g$} in an identical group *G*. The evaluation standard or criteria is that the finally selected best sensor $S_{best}$ should delegate all the sensors as much as possible. In other words, $S_{best}$ should be close to all the candidates in {$S_1$, $S_2$, …, $S_g$}. Therefore, we can pick out $S_{best}$ based on the evaluation condition in (9)-(11). Concretely, we expect that $S_{best}$ should be with the smallest sum of distances (denoted by $D_1$, $D_2$, …, $D_g$) with other sensors, i.e., {$S_1$, $S_2$, …, $S_g$} (see formulas (9)-(10)). Here, we define the distance between any two sensors $S_i$ and $S_j$, i.e., d($S_i$ , $S_j$) in (11). More specifically, for $S_i$ and $S_j$, we count the times that $C_{i,y} \ne C_{j,y}$ holds in total Y repetitions; if $C_{i,y} \ne C_{j,y}$ holds in many repetitions, then the distance between sensors $S_i$ and $S_j$ would be high.

9 $$\begin{aligned} S_{best}=\{S_{i}\vert D_{i} = Min \ \{D_{1},D_{2},...,D_{g}\}\} \end{aligned}$$

10 $$\begin{aligned} D_{i}=\sum \limits _{j=1}^{g}d(S_{i},S_{j}) \end{aligned}$$

11 $$\begin{aligned} d(S_{i},S_{j})=Count(C_{i,y} \ne C_{j,y}) \quad (1 \le y \le Y) \end{aligned}$$

Thus, through (9)-(11), we can finally pick out the best sensor $S_{best}$ from massive candidates in an identical group. Then in each group, the sensor $S_{best}$ is retained while the rest sensors are dropped. This way, we can maintain as few sensors as possible for a building which are used to monitor the environment information. As a result, the sensor deployment cost is reduced considerably, which significantly benefits the house owners involved in smart building.

More formally, the major procedure of FSPLO method is presented in Algorithm 1.

**Figure:**
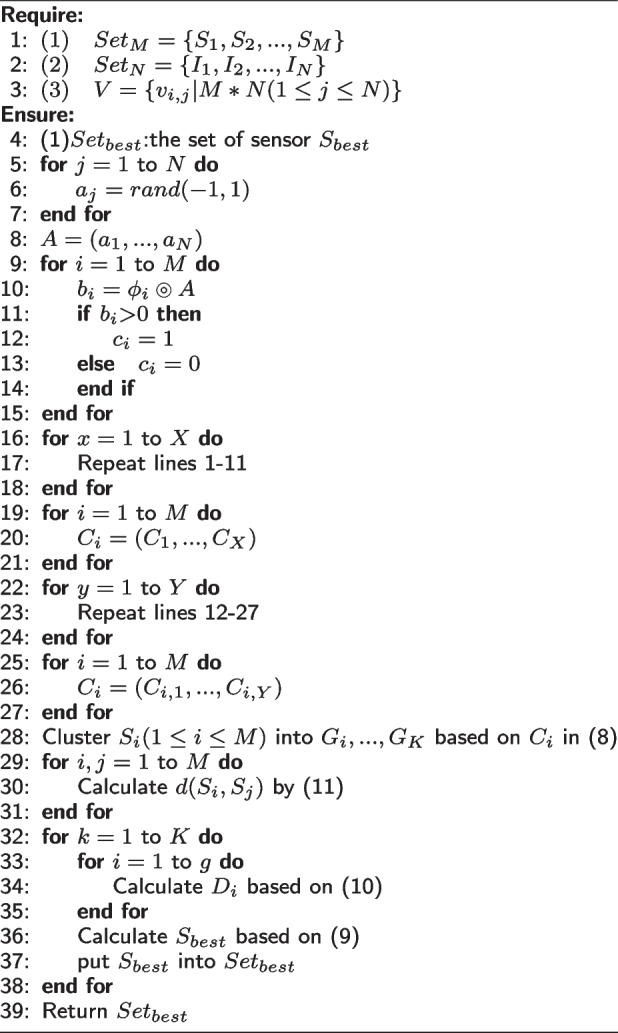
**Algorithm 1** FSPLO

## Experiment

### Experiment setup

**Datasets:** To validate the effectiveness of the FSPLO method proposed in this work, we have enacted massive simulated experiments based on a real world WSDREAM dataset. Concretely, this dataset contains 1,974,675 pieces of monitored data from 5825 objects and 339 users. We use the users and objects in the dataset to simulate the sensors and environment indicators, respectively. Thus, we can get a 339*5825 matrix *V* as in (1), i.e., parameters *M* = 339 and *N* = 5825.

**Baselines:** In addition, in the experiment comparisons, we compare FSPLO with another two baseline similarity calculation methods: UCF (User-based Collaborative Filtering) and ICF (Item-based Collaborative Filtering).

**Evaluation Scheme: ** The compared metrics in experiments include MAE (for measuring algorithm accuracy) and time cost (for measuring algorithm efficiency). We run each set of experiments 100 times to adopt their average performances.

**Experimental environment:** The proposed FSPLO and all the compared models are defined and trained on a Windows server with 3.10 GHz Intel I7-6500U CPU and 16.0 GB RAM, and implemented in Python 3.6.

### Experiment analysis

**Profile 1: Accuracy of FSPLO, UCF and ICF.**

**Fig. 3:**
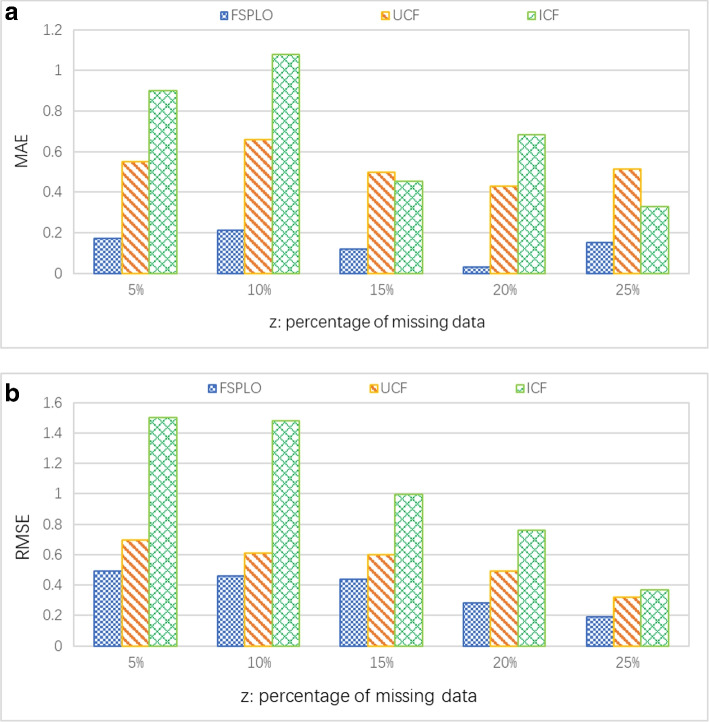
Accuracy comparison of FSPLO, UCF and ICF

In this profile, we observe the performances of three methods (i.e., FSPLO, UCF and ICF) in terms of accuracy (in the form of MAE and RMSE, smaller is better). To test the accuracy values, we remove some known data from the dataset and use the removed data to calculate the accuracy degree of different methods. Concretely, we vary the sparsity of matrix *V*, i.e., the percentage of removed or missing data (denoted by z) in the dataset from 5% to 25%; parameters *M* = 339 and *N* = 5825. Concrete running results are presented in Fig. 3. As the results in Fig. 3(a) report, the percentage z does not influence the MAE of three methods. Moreover, the MAE value of the proposed FSPLO method is always smaller than those of the rest two methods of UCF and ICF, which means that the accuracy of FSPLO method is the best among the three competitive methods. The reason is that we use LSH to search for the most similar sensors as well as their deployment places, while LSH has been proven an effective approximate neighbor discovery solution. Therefore, the accuracy of FSPLO method is often high, which means that FSPLO is effective to sensor volume reduction in green, economic and smart building. The RMSE comparison of the three methods is presented in Fig. 3(b) where the RMSE values of three methods all decrease with the growth of parameter z. Moreover, the RMSE value of our FSPLO is smaller than those of the rest two competitive methods. This also indicates that FSPLO achieves higher accuracy than other methods.

**Profile 2: Time cost of FSPLO, UCF and ICF.**

**Fig. 4:**
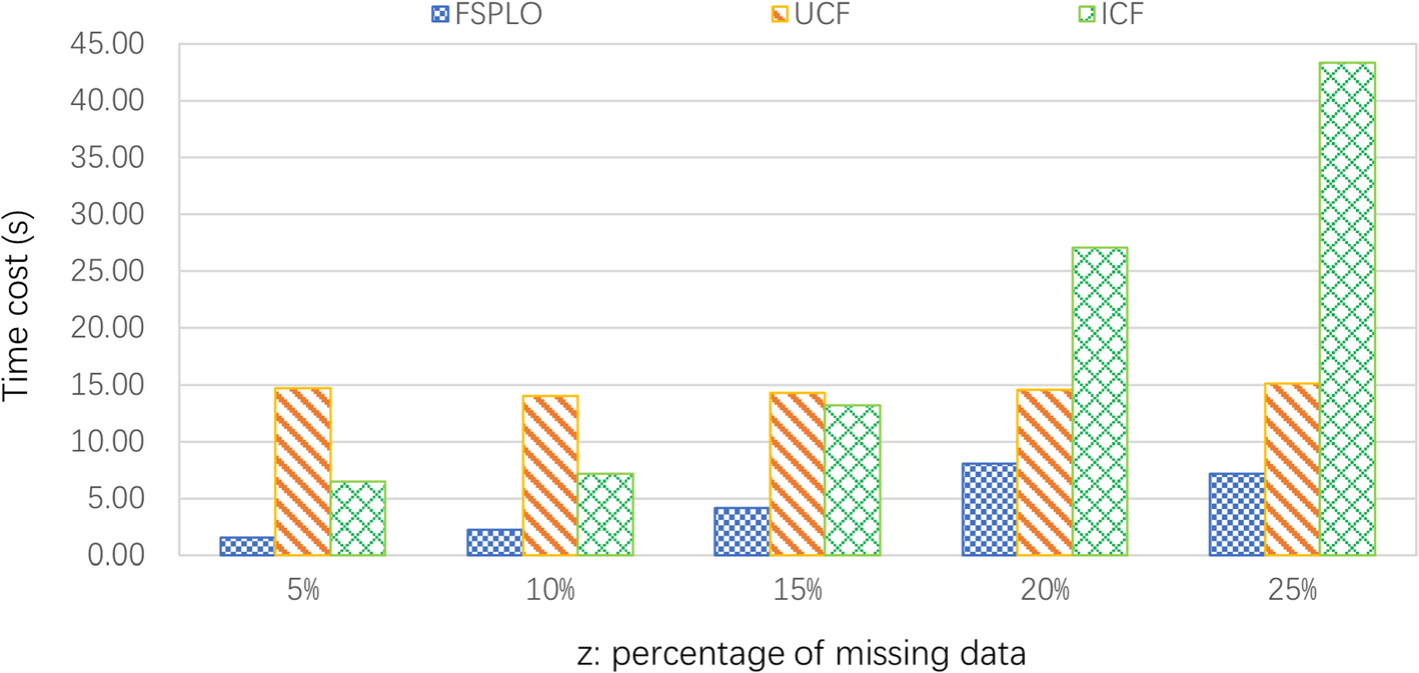
Time cost comparison of FSPLO, UCF and ICF

In this profile, we evaluate the time efficiency of three methods to show their respective applicable scope and extent in the big data environment. As introduced in Profile 1, we vary the percentage of removed or missing data (i.e., *z*) in the dataset from 5% to 25%; parameters *M* = 339 and *N* = 5825. Concrete running results are shown in Fig. 4. As Fig. 4 reports, the percentage *z* has direct impact on the time cost of three methods, because more computational time is needed to process more data in matrix *V*. Moreover, the MAE value of FSPLO method is always smaller than those of UCF and ICF, which indicates higher accuracy of FSPLO method compared to the other ones. The reason is like that of Profile 1: LSH has been proven an effective approximate neighbor discovery solution; therefore, FSPLO has the capability of discovering the really-similar sensors as well as their deployment places.

**Profile 3: Accuracy of FSPLO w.r.t. (** ***X, Y*** **) pair.**

**Fig. 5:**
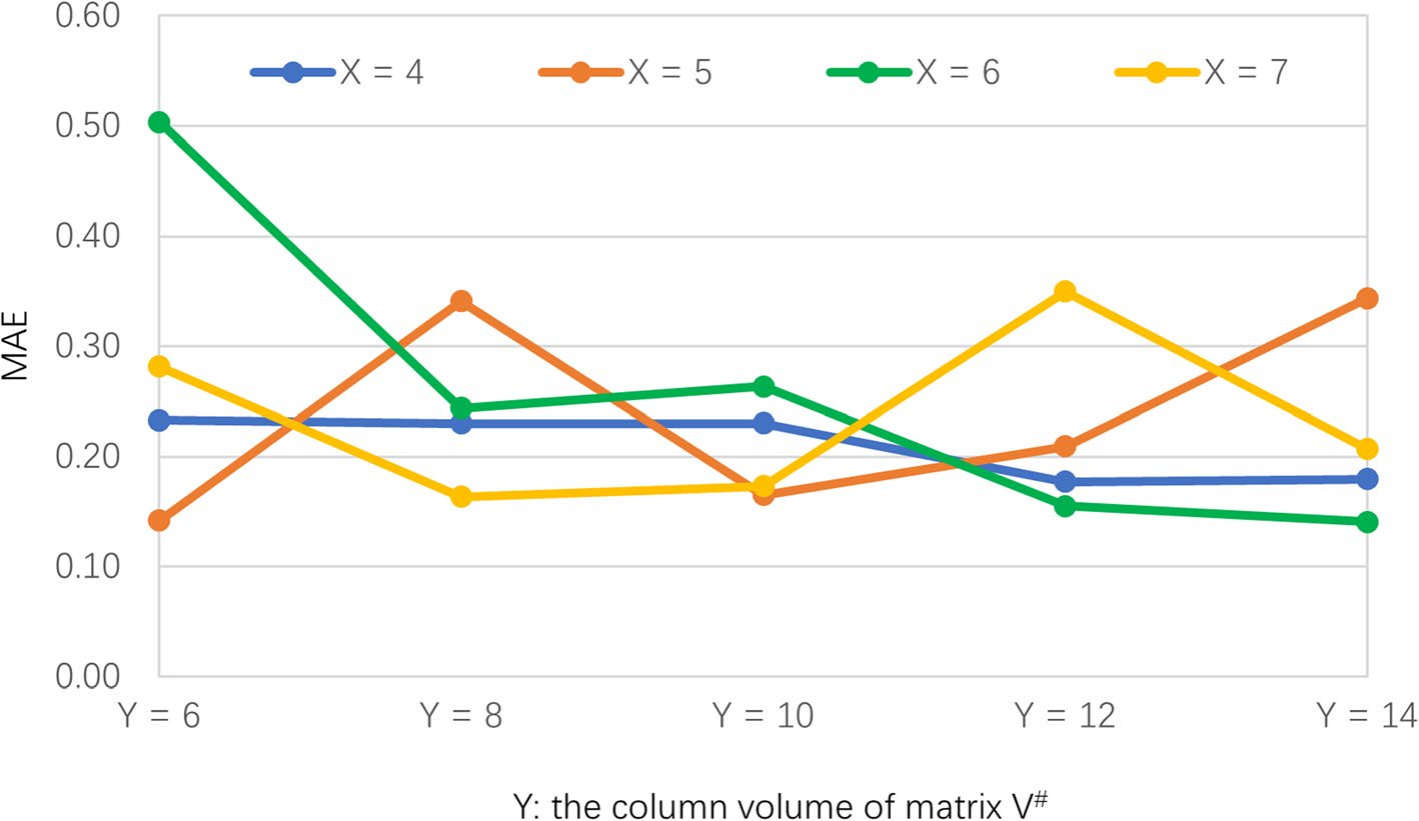
MAE of FSPLO w.r.t. (*X*, *Y*)

As Section 4 introduces, two parameters have played important roles in the performances of the proposed FSPLO method, i.e., *X* (i.e., the hashing times in Step 1) and *Y* (i.e., the column volume of matrix $V^\#$ in formula (7)). To observe their concrete influence towards the performance of FSPLO method, we design a set of experiments in which parameter *z* = 25%, *M* = 339, *N* = 5825, *X* is varied from 4 to 7, *Y* is varied from 6 to 14. We test the MAE values of FSPLO method, whose results are demonstrated in Fig. 5. As Fig. 5 shows, the MAE of FSPLO method does not render obvious variation tendency with respect to *M* or *N* directly, this is because these two parameters often influence the accuracy of FSPLO method together. Moreover, the accuracy is the lowest (i.e., MAE is the largest) when (*X*, *Y*) = (6, 6); the accuracy is the highest (i.e., MAE is the smallest) when (*X*, *Y*) = (5, 6) or (6, 14). This means that we need to jointly optimize the accuracy of FSPLO method by considering the two parameters simultaneously.

**Profile 4: Time cost of FSPLO w.r.t. (** ***X, Y*** **) pair.**

**Fig. 6:**
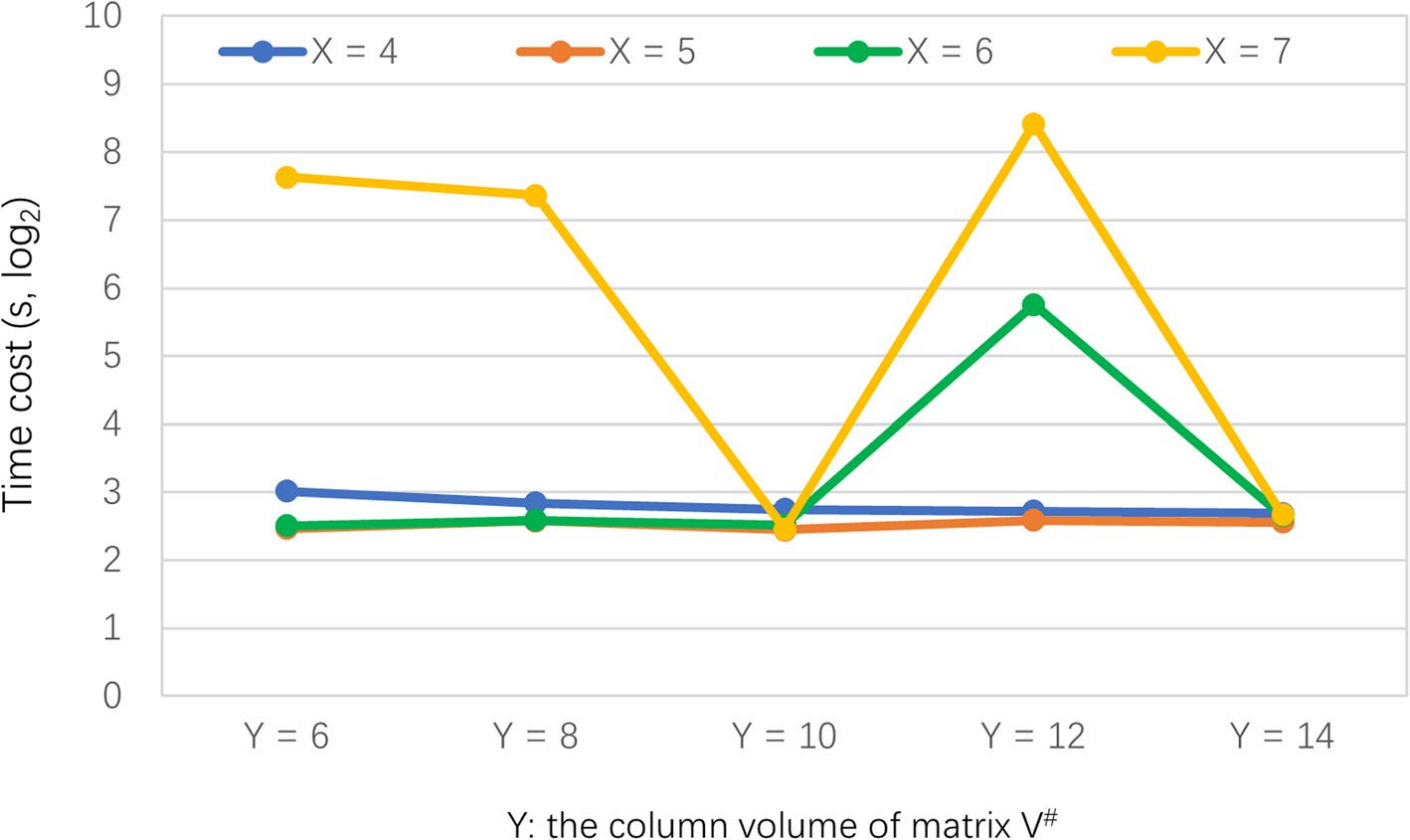
Time cost of FSPLO w.r.t. (*X*, *Y*)

In this profile, we observe the relationship between time cost and (*X*, *Y*) parameter pair. Parameter settings are as follows: *z* = 25%, *M* = 339, *N* = 5825, *X* is varied from 4 to 7, *Y* is varied from 6 to 14. Running results are reported in Fig. 6. As the results show, the time cost of FSPLO method is relatively stable with respect to the change of parameters *X* and *Y* (higher stability is most welcome for algorithms because we do not need to consider many parameters related to the algorithm). This is because the major step of FSPLO method, i.e., the Step 1 (i.e., sensor index creation) is often done in an offline way; therefore, the time cost of FSPLO method is often not related to these two parameters very much. However, as the results point out, the time cost is often the highest when parameter *X* = 7. This is because when *X* is higher, the neighbor search condition in stricter and consequently, it is possible that FSPLO method fails to discover similar sensors smoothly. In this situation, we need to repeat the algorithm multiple times to find out the similar sensors and cluster them correctly.

**Profile 5: Accuracy convergence of FSPLO.**

**Fig. 7:**
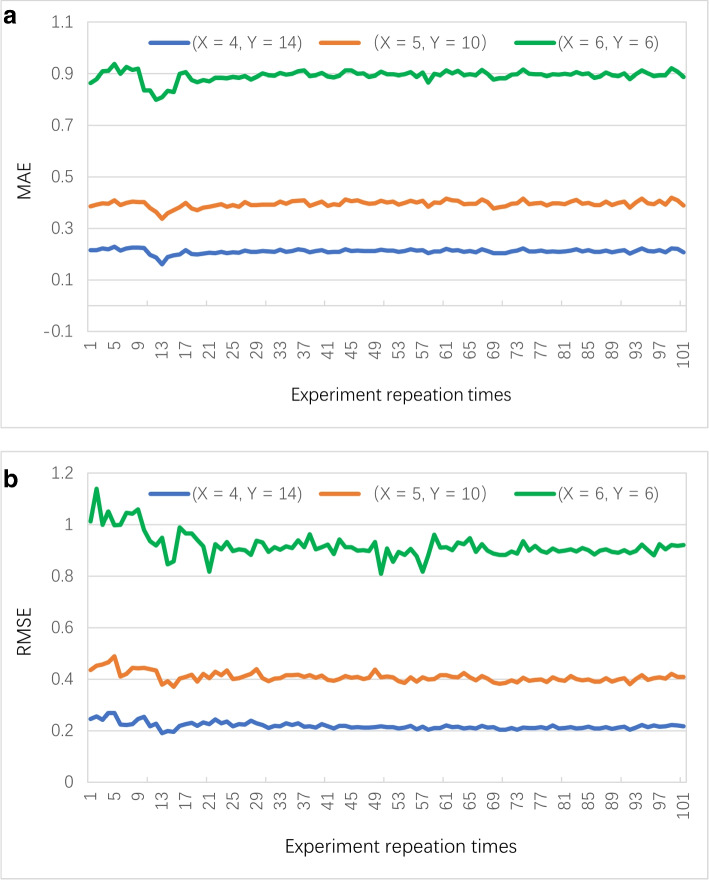
Accuracy convergence of FSPLO w.r.t. (*X*, *Y*)

In this profile, we test and observe the convergence of accuracy of our proposed FSPLO algorithm. Concretely, each set of experiments are repeated 100 times; *X* is varied from 4 to 6 while *Y* varies from 6 to 14. Experiment reports are presented in Fig. 7 where (a) and (b) denote the convergence of MAE and RMSE, respectively. As the results report, the accuracy of FSPLO is approximately stable when we repeat the experiments over 15 times (for MAE) and over 60 times (for RMSE). The stable convergence curves of MAE and RMSE indicates a good accuracy convergence of the proposed FSPLO.

## Conclusion

With the continuous development of human society, people are more interested in improving their living conditions, which calls for more economic, green and healthy buildings. In this situation, cloud-aided BIM has provided a promising way to optimize the design of smart buildings through various sensors deployed around the building. However, to accurately monitor the building information, we need to deploy many sensors which are often expensive enough and connect them to a central cloud platform. Therefore, it is necessary and challenging to minimize the sensor volume and save deployment cost. In view of this challenge, a hash-based fast sensor placement location optimization method (i.e., FSPLO) is proposed in this paper for BIM in cloud-aided smart building. Finally, we design a set of simulated experiments to prove the advantages of our proposed FSPLO method. The experimental results prove that our proposed method has higher performance performance than the existing methods in terms of accuracy and recommendation efficiency.

However, FSPLO method only considers statistic sensor data of buildings. However, sensor data are often time-aware and influenced dynamically in various smart applications in Internet of Things. Therefore, in the future, we will refine FSPLO method by incorporating the time factor. In addition, many parameters are existed in FSPLO method and their concrete parameter values are difficult to determine. In the future, we will investigate to calculate the optimal parameter values in FSPLO through various Artificial Intelligence technologies including Deep Learning [32], Neural Networks [33], Transfer Learning, Federated Learning and so on. At last, the data formats or types in big data applications are often multiple. Therefore, we will further refine our work by considering the diversity of data format or types in future.

## Data Availability

https://github.com/wsdream.

## Abbreviations

LSH: Locality-Sensitive Hashing
BIM: Building Information Modeling
FSPLO: Fast Sensor Placement Location Optimization
